# Assessment of reliability and validity of a handheld surface spine scanner for measuring trunk rotation in adolescent idiopathic scoliosis

**DOI:** 10.1007/s43390-023-00737-3

**Published:** 2023-07-26

**Authors:** Jack Z. Wei, Berry K. C. Cheung, Sunny L. H. Chu, Parker Y. L. Tsang, Michael K. T. To, Johnson Y. N. Lau, Kenneth M. C. Cheung

**Affiliations:** 1 Department of Orthopaedics and Traumatology, The University of Hong Kong, Pok Fu Lam, Hong Kong SAR China; 2 Avalon SpineCare (HK) Ltd., Hong Kong, Hong Kong SAR China

**Keywords:** Adolescent idiopathic scoliosis, Trunk rotation, Surface scanner, Scoliometer

## Abstract

**Purpose:**

To assess the reliability and validity of a handheld scanner (SpineScan3D) for trunk rotation measurement in adolescent idiopathic scoliosis (AIS) subjects, as compared with Scoliometer.

**Methods:**

This was a cross-sectional study with AIS subjects recruited. Biplanar spine radiographs were performed using an EOS imaging system with coronal Cobb angle (CCA) determined. The angle of trunk rotation (ATR) was measured using Scoliometer. SpineScan3D was employed to assess the axial rotation of subjects’ back at forward bending, recorded as surface tilt angle (STA). Intra- and inter-examiner repeats were conducted to evaluate the reliability of SpineScan3D.

**Results:**

97 AIS patients were recruited. Intra- and inter-examiner reliability of STA measures were good to excellent in major thoracic and lumbar curves (p < 0.001). A strong correlation was found between STA and ATR measures in both curve types (p < 0.001) with a standard error of the ATR estimate of between 1 and 2 degrees from linear regression models (R squared: 0.8–0.9, p < 0.001). A similar correlation with CCA was found for STA and ATR measures (r: 0.5–0.6, p < 0.002), which also demonstrated a similar sensitivity (72%-74%) and specificity (62%-77%) for diagnosing moderate to severe curves.

**Conclusion:**

SpineScan3D is a handheld surface scanner with a potential of wide applications in subjects with AIS. The current study indicated that SpineScan3D is reliable and valid for measuring trunk rotation in AIS subjects, comparable to Scoliometer. Further studies are planned to investigate its measurements in coronal and sagittal planes and the potential of this device as a screening and monitoring tool.

**Trial registration number (date of registration):**

HKUCTR-2288 (06 Dec 2017).

**Level of evidence:**

Level III.

## Introduction

Adolescent idiopathic scoliosis (AIS) is a three-dimensional spinal deformity, characterized by a sideway curvature, known as Cobb angle, of over ten degrees [1]. With the aetiology and pathogenesis remaining largely unknown, it affects two to three percent of the adolescent population, more commonly seen in girls than boys [2]. If left untreated, subjects with AIS are at risk of curve progression during puberty [3], while severe curves may lead to impaired lung function, severe back pain, unsightly appearance, and psychological impacts on affected children [4]. Therefore, early detection and close monitoring are essential for managing AIS.

Although the value of screening for the early detection of AIS and the efficacy of bracing for preventing curve progression have been well established [2, 5], repeated radiation exposure remains a major concern in AIS management [6]. Unfortunately, for children diagnosed with mild-to-moderate scoliosis, repeated radiographic assessments are routinely performed during their puberty at an interval of six to twelve months to closely monitor the scoliotic spine [7], until the need of invasive surgical correction for severe curves. A recent study by Mehta et al. has demonstrated the cumulative effect of radiation throughout the course of treatment in scoliosis children, while significantly higher dose exposure was reported in patients with surgical treatment, as compared with the conservatively treated cohort, leading to a significantly higher estimated risk of cancer induction [8]. Despite the application of low-dose slot scanning with the EOS imaging system, research efforts have been focused on reducing the reliance on radiographs for AIS management by applying non-invasive techniques, including surface topography [9–11] and ultrasound [12, 13], while handheld devices have been developed to enhance the portability and efficiency of scoliosis assessments [14–16].

Scoliometer is one of the earliest attempts to provide a radiation-free method for assessing scoliosis [17]. It is a handheld inclinometer that quantifies the surface rotational prominence of the subject’s back at a forward bending posture, recorded as an angle of trunk rotation (ATR). Studies have demonstrated the reliability of this tool and its moderate correlation with radiographic coronal Cobb measurements [18]. Together with the good sensitivity and specificity in indicating scoliosis, Scoliometer has become one of the main tools in the scoliosis screening programs, which also include clinical signs and the Moiré topography [5, 19].

Despite its wide applications in scoliosis management, there are several limitations for further development. First, Scoliometer measures axial trunk rotation at forward bending driven by the gravity, while the axial trunk rotation at other postures, such as standing posture, can not be measured, which can provide more information about the trunk to reflect the spine at a natural posture. Second, Scoliometer can only assess the trunk in the axial plane of the spine, while the coronal and sagittal profiles are not involved, despite the fact that scoliosis is a three-dimensional deformity. Third, ATR measured by Scoliometer is normally recorded at the apex of each back hump in thoracic and lumbar regions, but the exact locations and non-apical ATR readings of the back humps are not recorded, which can provide more information about the pattern of the scoliosis. Last, although Scoliometer is easy to use, there is a risk of human errors during data transcription and transposition due to the reliance on manual reading and data entry, especially in scoliosis screening of a large scale and remote uses by guardians in the community.

A novel handheld device with a low manufacturing cost (current estimated cost in 2023: 64 US dollars), known as SpineScan3D (Avalon SpineCare (HK) Ltd., Hong Kong), was developed for the assessment of surface topography. With the built-in electronic sensors and a user-friendly smartphone software, the device can reduce the reliance on manual ATR reading and data transcription and potentially allow remote uses in the community and at home by guardians, facilitating the early screening and closer monitoring of AIS.

The purpose of this study is to verify the reliability and validity of the novel surface scanner for measuring trunk rotation in AIS subjects. We hypothesize that the device is reliable and valid in trunk rotation measurements, comparable to Scoliometer.

## Methods

AIS subjects aged 10 to 17 were recruited at a scoliosis clinic. Subjects with the following conditions were excluded: (1) scoliosis of other causes, (2) leg length discrepancy, (3) history of spinal surgeries, (4) stand unsteadily at forward bending posture during measurements. Biplanar spine radiographs were taken as a clinical routine using an EOS imaging system (EOS imaging, France) with coronal Cobb angle (CCA) measured on digitized films. Based on the spinal level of the apical vertebrae of the major curve, recruited subjects were divided into a thoracic group (T2-T12) and a lumbar group (L1-L4). For surface assessments, a 90-degree forward bending posture at standing was adopted with the subject’s back exposed from C7 to L5. Scoliometer measurement was performed by a single well-trained researcher, who was a medical graduate with 3-year experience in the measurement trained by an orthopaedic surgeon. During the measurement, Scoliometer was manually moved along the spinous processes from C7 to L5 on the back in contact with the paraspinal skin surface. Maximum ATR readings and the sidedness were manually determined and recorded for back prominence in thoracic and lumbar regions, also known as rib and loin humps [17].

**Fig. 1:**
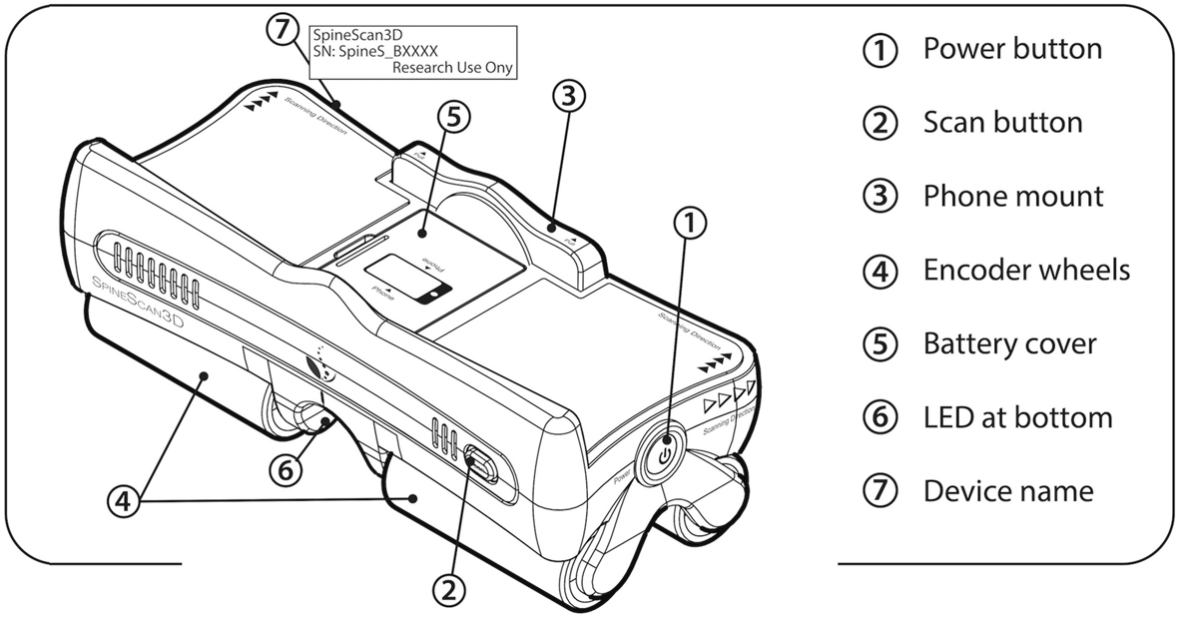
Schematic of SpineScan3D device construct

SpineScan3D device was constructed with an outer plastic cart with four wheels and a spring-loaded platform, two encoders inside the wheels, and an inner electronic chip embedded with a gyroscope and an accelerator (Fig. 1), which are motion sensors that have been widely used in different electronics like smartphone and wearables, and medical device for gait analysis and rehabilitation [20, 21]. During the measurement, as the device moving along subject’s back, the three-dimensional tilting angles of the device were automatically captured by the sensors every 0.1 s. A customized mobile application was developed with a connection to the device via Bluetooth for providing a user interface and instant results on the smartphone mounted on the device. Collected data was automatically stored in the local mobile application and uploaded to a remote cloud server via Wi-Fi.

**Fig. 2:**
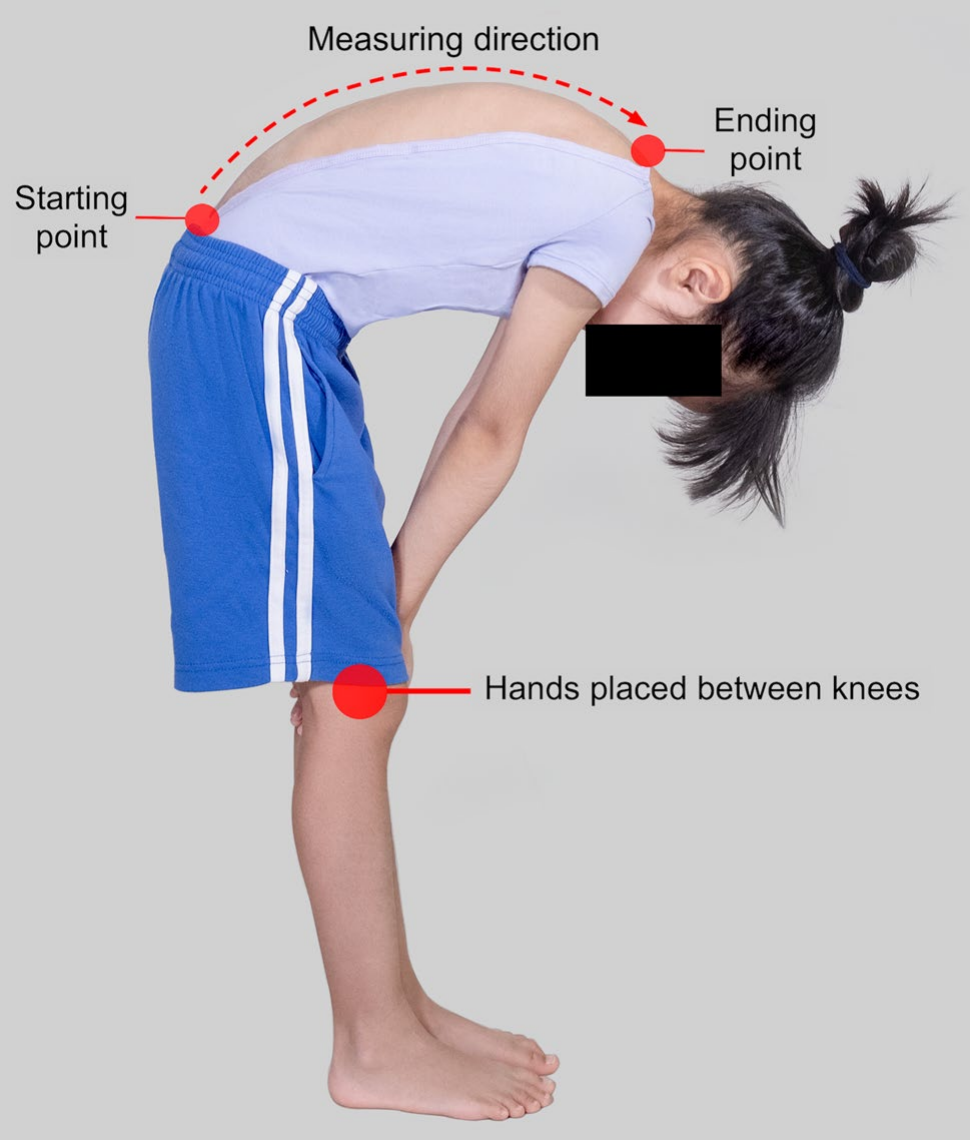
Image of a subject taking a forward bending posture with her feet at shoulder width, hands clasped and placed between the knees, and both arms and knees keeping straight for SpineScan3D measurement

**Fig. 3:**
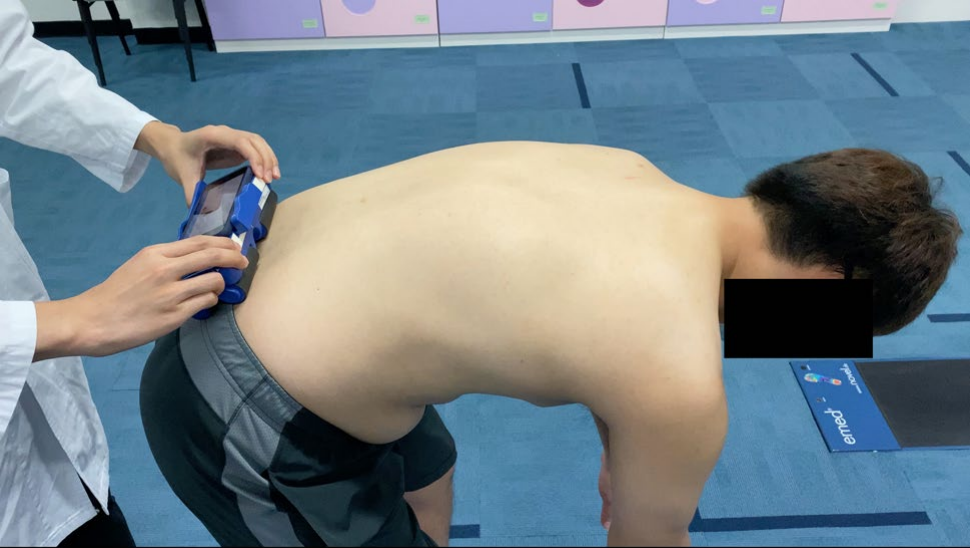
Image of the SpineScan3D device placed on the back of a subject by an examiner standing behind

For SpineScan3D measurement, given the fact that tested subjects might experience an itchy feeling on their back during scanning, additional preparations were carried out to eliminate the body movement during scanning. First, the subject's posture was standardized with their feet at shoulder width, hands clasped and placed between the knees, and both arms and knees kept straight (Fig. 2). Second, trial scans were conducted to eliminate the itchy feeling on subject’s back due to the wheels of the device rolling on the skin surface. Last, to reduce the impact of subject’s itchy feeling on the reliability of the measurement, repeat scans were conducted when the subject could not stand steadily and moved the body during the measurement. During the assessment, with the examiner standing right behind the subject (Fig. 3), the device was first placed on the back at three to four centimetres below the intercristal line (L4) as the starting point (L5) and then moved by the examiner toward the spinous process of C7 as the ending point, following the central line of the back. During the scanning, based on the nature of the sensor reading every 0.1 s, the device was maintained at a moving speed between 10 to 15 cm per second by the examiner, while manual forces on the skin were avoided to reduce the impact of soft tissue on the reliability of the measurements.

In this study, the surface contour of the back was assessed in the axial plane using the device, recorded as surface tilt angle (STA), which was the maximum tilt angle of rib or loin humps, equivalent to ATR readings from the Scoliometer. To assess the reliability of the device, for each subject, SpineScan3D measurements were conducted by two independent examiners and repeated by the first examiner at five minutes apart.

Statistical analysis was conducted using SPSS Statistics, version 27.0 (IBM Corp., Armonk, N.Y., USA). Continuous variables were described as mean and standard deviation (SD). Intraclass correlation coefficient (ICC) (two-way mixed model, single measure) and Bland–Altman plot were used to evaluate the intra- and inter-examiner reliability and agreement. Pearson correlation coefficient and linear regression analysis were applied to assess the validity of STA measures from SpineScan3D, as compared with ATR readings from the Scoliometer, which was further confirmed by comparing their correlation with CCA from radiographs. The results of reliability and validity were interpreted based on the following criteria: 0.00 to 0.29 as very low, 0.30 to 0.49 as low, 0.50 to 0.69 as moderate, 0.70 to 0.89 as high and 0.90 to 1.00 as very high [22]. As the current study was the first to validate the device, a post-hoc power analysis was performed to assess the achieved power of the detected correlation between STA and ATR, and the correlations of STA and ATR with CCA. Receiver operating characteristic (ROC) curve analysis was performed to compare the diagnostic accuracy of both tools in predicting moderate to severe curves (CCA ≥ 20 degrees). Statistical significance was considered at a p value below 0.05.

## Results

Ninety-seven AIS subjects were recruited at a mean age (year) of 14 with 63 females and 34 males. Based on the spinal level of apical vertebrae of the major curve, 53 and 44 subjects were recruited in the thoracic and lumbar subgroups, respectively. The results of curve magnitude and surface measurements were summarized in Table 1.

**Table 1:** Descriptive results of radiographs, Scoliometer and SpineScan3D measurements

	Measures	Mean	Standard Deviation	Minimum	Maximum
Major thoracic curves	CCA (degree)	25.1	10.4	10.6	73.3
	ATR (degree)	8.6	3.9	3.0	23.0
	STA (degree)	8.0	3.6	2.8	18.2
Major lumbar curves	CCA (degree)	24.5	8.2	12.3	40.6
	ATR (degree)	9.0	3.5	2.0	17.0
	STA (degree)	10.0	4.0	3.1	20.1

*CCA* coronal Cobb angle, *ATR* angle of trunk rotation, *STA* surface tilt angle

**Fig. 4:**
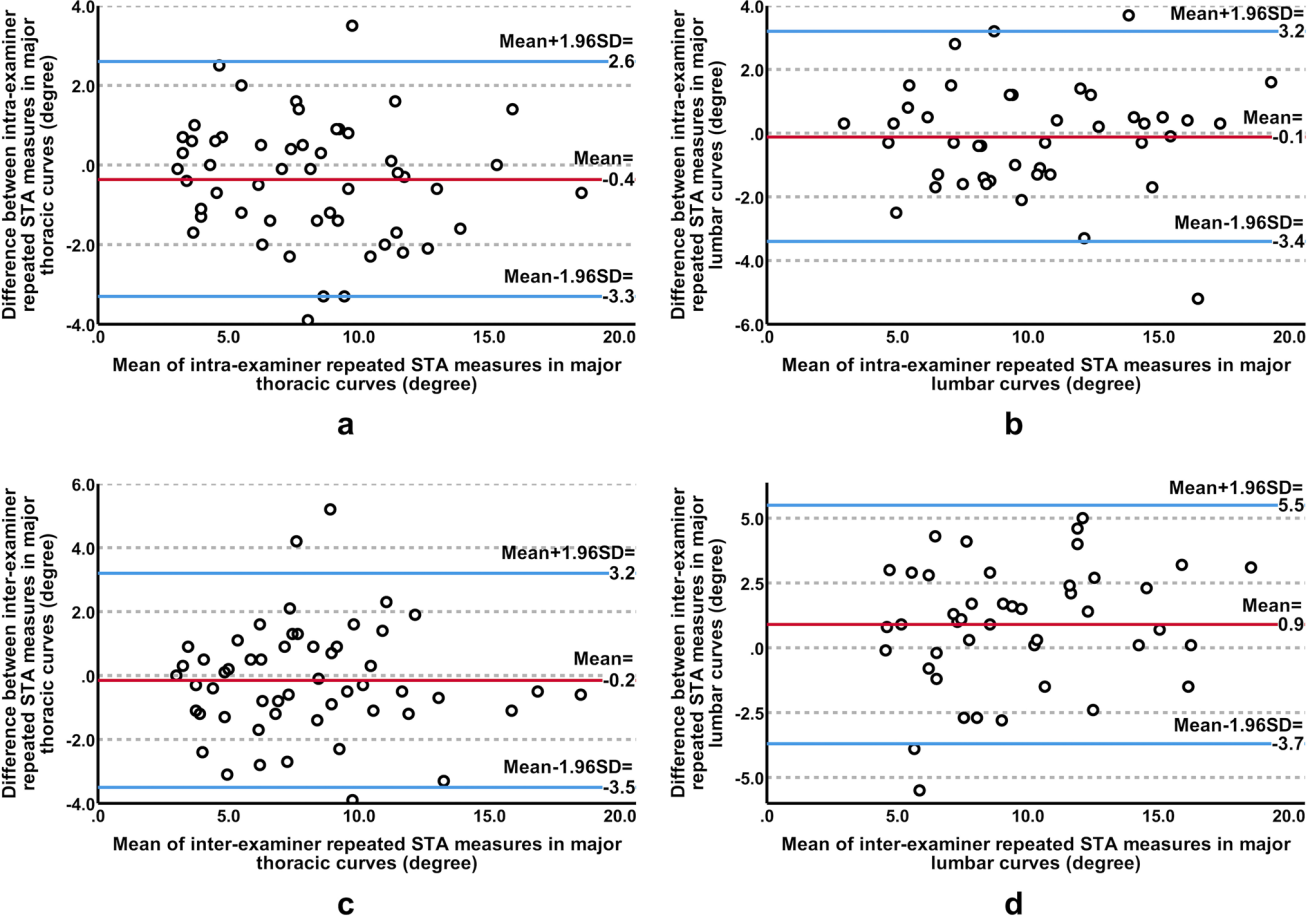
Bland–Altman plots of intra-examiner (**a, b**) and inter-examiner (**c, d**) repeated surface tilt angle (STA) measures in major thoracic (**a, c**) and major lumbar (**b, d**) curves

The intra-examiner reliability of STA measurements was high to very high in major thoracic curves (ICC: 0.9, 95% confidence interval (CI) 0.86–0.95, p < 0.001) and major lumbar curves (ICC: 0.9, 95% CI 0.85–0.95, p < 0.001). A high inter-examiner reliability was also observed in measuring major thoracic curves (ICC: 0.9, 95% CI 0.81–0.93, p < 0.001) and major lumbar curves (ICC: 0.8, 95% CI 0.63–0.88, p < 0.001). The agreement of intra- and inter-examiner repeats was shown in Bland–Altman plots in Fig. 4. The difference between repeated STA measures was not statistically significant (p > 0.05), except for inter-examiner repeats in major lumbar curves (mean difference: 0.9 degrees, SD: 2.4 degrees, p < 0.05).

**Fig. 5:**
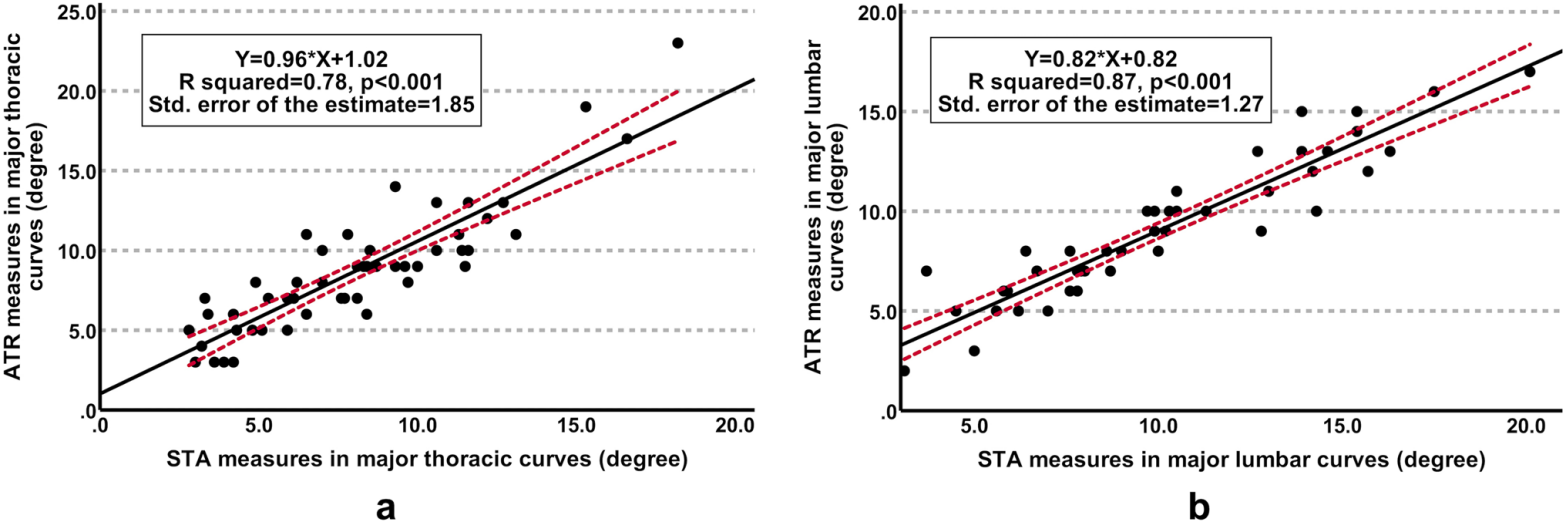
Linear regression analysis of surface tilt angle (STA) for predicting the angle of trunk rotation (ATR) in major thoracic (**a**) and major lumbar (**b**) curves

Table 2 showed the Pearson correlation analyses. High to very high correlations were observed between STA and ATR measures in major thoracic (r: 0.9, p < 0.001) and major lumbar curves (r: 0.9, p < 0.001). A good fit linear regression model was detected for both curve types (R squared: 0.8–0.9, p < 0.001) with a standard error of the ATR estimate of between one and two degrees, respectively (Fig. 5). As compared with CCA measured from radiographs, similar correlations were found for STA and ATR measures in major thoracic (r: 0.6, p < 0.001) and major lumbar curves (r: 0.5, p < 0.002). Power analysis was conducted to calculate the achieved power using G*Power (version 3.1.9.6) [23]. With the sample size of 97 in the current study, the achieved power of detecting the bivariate linear correlations of 0.5 to 0.9 ranged from 99.9% to 100% at an alpha level of 0.05.

**Table 2:** Pearson correlation coefficient between surface tilt angle (STA), angle of trunk rotation (ATR) and coronal Cobb angle (CCA)

		Pearson’s r	p value
Major thoracic curves	STA vs. ATR	0.9	0.001
	STA vs. CCA	0.6	0.001
	ATR vs. CCA	0.6	0.001
Major lumbar curves	STA vs. ATR	0.9	0.001
	STA vs. CCA	0.5	0.002
	ATR vs. CCA	0.5	0.002

*CCA* coronal Cobb angle, *ATR* angle of trunk rotation, *STA* surface tilt angle

The results of the ROC curve analysis were provided in Table 3. Similar sensitivity (72%-74%) and specificity (62%-77%) were observed for STA and ATR measures to predict a CCA ≥ 20 degrees in major thoracic and major lumbar curves, using cut-off values of 6.8 to 7.9 degrees.

**Table 3:** Receiver operating characteristic (ROC) curve analysis of surface tilt angle (STA) and angle of trunk rotation (ATR) for predicting a coronal Cobb angle ≥ 20 degrees

	Measures	Area under the ROC curve	Cut-off value (degree)	Sensitivity	Specificity
Major thoracic curves	STA	0.7	6.8	72%	70%
	ATR	0.8	7.5	72%	77%
Major lumbar curves	STA	0.7	7.9	74%	62%
	ATR	0.7	7.5	74%	69%

## Discussion

AIS is the most common spinal deformity in adolescents with a known risk of curve progression during bone growth, which may end up with noticeable body asymmetries and a need for surgical correction. The current practice of school screening programs and regular clinic follow-up for timely bracing care has drawn increasing attention related to repeated radiation exposure in the affected population [24, 25]. Scoliometer has been actively involved as a handheld and non-radiographic tool for scoliosis screening and assessing treatment outcomes, which however has inherent limitations of reflecting three-dimensional profiles of the spine, screening large-scale at-risk populations efficiently, and avoiding potential human errors during manual documentation.

SpineScan3D is a handheld non-invasive device with a low manufacturing cost, which has built-in electronic sensors for automatically detecting surface profiles three-dimensionally and a user-friendly smartphone interface for data storage locally and remotely. Different from the Scoliometer and other handheld devices [14–16, 26], the device can reduce the reliance on manual data reading and transition, thus having a potential of widespread uses within the community by guardians both locally and remotely, which helps facilitate the early screening and closer monitoring of scoliosis. Moreover, Scoliometer can only measure the trunk in axial plane, while SpineScan3D has the potential of measuring the trunk in three dimensions, which can provide more insights about the three-dimensional profiles of the deformity that have been recently proven to help predict the progression of scoliosis [27, 28]. This study aimed to validate this tool against the Scoliometer for measuring trunk rotation in AIS.

The current study demonstrated high to very high reliability of SpineScan3D for measuring rib and loin humps at forward bending, comparable to the reliability of Scoliometer as reported in the literature [22]. As compared with the Scoliometer which involves manual work on following the spinous process and recording readings of trunk rotation, SpineScan3D measurement was conducted following the midline of the back with sensor readings automatically saved, calculated, and with data presented on a smartphone. A wheel-based design was adopted to facilitate its movement on the skin surface, enhance the device’s stability during measurement and allow the potential localization of the back prominences. Like any other contact-based tools for profiling surface contour, measuring errors remain a major concern [15, 26, 29], which attributes to factors including skin tissue thickness, device positioning, examiner skills, and subject’s movement during the assessment. Nevertheless, the overall reliability results demonstrated in this study showed that the impact of contact-related factors was not significant, and the results were very reliable. Regarding the potential need for repetition and exclusion due to unstable posture, it only occurred in less than 5% of all the tested subjects in the current study. The authors believe that the extra time consumed for repeated measurements was not significant, as this is a handheld electronic device with a user-oriented interface and thus the ability of large-scale assessments in a short period of time.

In terms of the validity analysis, despite the differences in device designs and measuring protocols, a high correlation was found between SpineScan3D and Scoliometer in trunk rotation measurement, while the correlation with radiographic coronal Cobb measures was comparable between the two techniques. Moderate correlations were detected between axial trunk rotation measured by SpineScan3D and radiographic coronal Cobb measures in both thoracic and lumbar regions, similar to the validity of Scoliometer demonstrated in previous studies [22, 30]. Although recent studies on advanced rasterstereography and depth camera assessment tools have found moderate-to-high correlations between surface topography measurements and radiographic Cobb measurements [9, 16], wider applications of these tools in both clinic and community basis were restricted due to the poor portability and the relatively higher manufacturing cost. The clinical validity of SpineScan3D was further proven by its moderate-to-high diagnostic accuracy (sensitivity: 72%-74%, specificity: 62%-70%) for a moderate-to-severe curve, comparable to Scoliometer (sensitivity: 72%-74%, specificity: 69%-77%), which agreed with the accuracy of Scoliometer alone for detecting a scoliosis ≥ 20 degrees in a previous study by Lee et al. [31]. As compared with ultrasound-based tools [13], the overall screening accuracy of SpineScan3D was comparable, although ultrasound-based techniques showed a high sensitivity but a lower specificity. Future studies are required to further assess the accuracy of the device for detecting a curve ≥ 10 degrees in a prospective population-based screening study.

There are a few limitations of the present study. First, coronal and sagittal surface profiles of the back were not yet studied. We are planning to further study and validate this device for assessing three-dimensional surface contour at a standing posture. Second, the ability of SpineScan3D for localizing rib and loin humps on the back and subsequently predicting different curve patterns was not yet verified. Third, STA measures were only evaluated for major humps in this study, although the value of minor humps and mathematical formulas in predicting CCA has been suggested in previous studies [32, 33].

In conclusion, we showed that SpineScan3D is reliable and valid in measuring trunk rotation in AIS subjects, and comparable to Scoliometer. With less involvement of manual input and less reliance on examiners’ expertise and experience, it has a great potential for broad use in both clinical and community settings and on a local or remote basis. We plan to soon assess its accuracy in three-dimensional measurements and performance in scoliosis screening and progression monitoring, and these results may provide more insights into the broader applicability of this device.

## Data Availability

The datasets generated and analyzed in the current study are available from the corresponding author upon request.
